# Comparing volume-clamp method and intra-arterial blood pressure measurements in patients with atrial fibrillation admitted to the intensive or medium care unit

**DOI:** 10.1007/s10877-017-0044-9

**Published:** 2017-07-07

**Authors:** G. F. N. Berkelmans, S. Kuipers, B. E. Westerhof, A. M. E. Spoelstra-de Man, Y. M. Smulders

**Affiliations:** 1 Department of Vascular Medicine, University Medical Center Utrecht, PO Box 85500, 3508 GA Utrecht, The Netherlands; 2 Department of Internal Medicine, Zaans Medical Center, Zaandam, The Netherlands; 3 Department of Pulmonary Diseases, VU University Medical Center, Amsterdam, The Netherlands; 4 Department of Intensive Care, VU University Medical Centre, Amsterdam, The Netherlands; 5 Department of Internal Medicine, VU University Medical Center, Amsterdam, The Netherlands; 6 Laboratory for Clinical Cardiovascular Physiology, Center for Heart Failure Research, Academic Medical Center, Amsterdam, The Netherlands

**Keywords:** Atrial fibrillation, Blood pressure, Noninvasive measurement, Validation, Volume clamp method

## Abstract

International guidelines highlight the importance of blood pressure (BP) in patients with atrial fibrillation (AF). However, BP measurement in AF is complicated by beat-to-beat fluctuation. Automated BP measurement devices are not validated for patients with AF and no consensus exists on how to measure BP in AF manually. Beat-to-beat BP measurement using the volume-clamp method (VCM) could represent a non-invasive method to accurately assess BP, but has not been validated in AF. 31 admitted patients with sustained AF and 10 control patients with sinus rhythm underwent simultaneous intra-arterial and non-invasive BP measurement using a VCM monitor (Nexfin^®^, BMEYE, Amsterdam, The Netherlands). Patients with compromised peripheral perfusion, high doses of vasopressor drugs or peripheral edema were excluded. Differences in systolic, diastolic and mean BP of 5 (standard deviation; SD 8) mmHg (accuracy and precision) between both methods were considered acceptable. Additionally, the magnitude of beat-to-beat fluctuations in systolic BP of both methods was compared. In AF, the differences between noninvasive and invasive BP were −4 (SD 12), +1 (SD 7) and 0 (SD 8) mmHg for systolic, diastolic and mean BP respectively. Absolute differences in beat-to-beat BP fluctuations were 1.5 (IQR 0.8–3.8) mmHg. Accuracy of VCM in AF was similar to sinus rhythm. In conclusion, in patients with AF, accurate and precise measurement of non-invasive beat-to-beat BP measurement using the VCM is possible, the one exception being the precision of systolic BP. Beat-to-beat variability can be accurately reproduced.

**Electronic supplementary material:**

The online version of this article (doi:10.1007/s10877-017-0044-9) contains supplementary material, which is available to authorized users.

## Introduction

Atrial fibrillation (AF) is the most common arrhythmia with an incidence of 60 per 100,000 person-years in women and 78 per 100,000 person-years in men. The prevalence increases with age from 0.1% among adults younger than 55 years to 9.0% in persons aged 80 years or older [1] and the life-time risk of AF is ~16%. AF is associated with an increased risk of death and cardiovascular disease [2]. Reduction of cardiovascular risk thus has priority in patients with AF.

Globally, elevated blood pressure (BP) is the leading risk factor for death and disability-adjusted life-years lost [3], but is also an important risk factor for AF [4]. In patients with AF, hypertension is prognostically relevant [5], affecting both thromboembolic and bleeding risk [6, 7]. Assessment of BP in AF is, however, complicated due to variation in BP with each heartbeat. Scarce data indicate that BP measurements in patients with AF is associated with a greater variability compared to sinus rhythm (SR) [8].

Given the importance of BP in patients with AF, the subject of accurate measurement receives surprisingly little attention in the international literature. Current AF guidelines do not provide clear or uniform recommendations on how to measure BP in AF [9, 10]. No consensus method exists for manual BP measurement in patients with AF, and manual methods have not been compared to a reference method, such as intra-arterial measurement [11]. Automatic oscillometry, the most commonly used method of BP assessment in routine clinical care, is not validated for BP measurement in AF according to international standards. In a few studies, automatic oscillometers have been compared to conventional manual auscultatory measurements [12, 13] which, as just mentioned, are themselves inaccurate and not validated for AF.

Taken together, BP monitoring in patients with AF remains problematic. Intra-arterial measurements reliably follow rapid changes in BP, even on a beat-to-beat basis, and are the preferred reference method for BP measurement in AF. However, this is obviously unfeasible in daily clinical practice given the invasive nature of the procedure. Reliable non-invasive beat-to-beat BP measurement would be ideal to serve as either a routine clinical method, or as a reference method for comparison of other non-invasive methods, such as manual or oscillometric measurements.

The volume-clamp method (VCM) is a non-invasive technique that has been validated in several different populations and conditions (subjects with a wide range of BPs from hypotensive to hypertensive, cardiothoracic surgery patients and children) [14–20]. Arterial blood volume of the finger is measured by an optical plethysmograph, mounted in an inflatable finger cuff, and clamped by a fast control system [21, 22]. To date, the VCM method has not been tested in patients with AF. Conceivably, it may be less reliable in AF due to substantial beat-to-beat variability. We performed this study to assess the accuracy of non-invasive beat-to-beat BP measurement with the VCM method by comparing it to intra-arterial measurement as the reference method.

## Methods

### Study population

For this study, the need to obtain informed consent of the participating patients was waived by the Medical Ethical Committee. Patients with AF who had an intra-arterial catheter in the radial artery for BP monitoring on the intensive care unit, medium care unit or coronary care unit were eligible for this study. Patients with reduced peripheral perfusion as assessed by clinical features (cold, pale hands and fingers), high-dose vasopressor drugs (>0.24 µg/kg/min norepinephrine) and/or moderate or more severe peripheral edema [21] were excluded, as well as agitated patients (VCM is affected by rapid movement of the hand). For comparison, similar data and analyses were obtained from ten patients from the same departments with SR.

### Procedures

A finger cuff was applied to the mid-phalanx of the second, middle or fourth finger ipsilateral to the arm with the intra-arterial catheter. The “finger side” of the heart reference system (HRS) was fixed next to the finger on which the cuff was applied. The “heart side” of the HRS was fixed at right atrial level on the patient’s gown. The level of the right atrium was assumed to be located at the fourth intercostal space at the mid-anterior–posterior diameter of the chest wall. At the same height, the pressure transducer was positioned on a pole next to the patient’s bed, preventing errors in BP due to difference in height [21, 22].

All patients included had an intra-arterial line for BP monitoring as part of usual care. The arterial line was placed in the radial artery (left or right) with a 20 gauge cannula. The catheter was connected to a monitor of Philips IntelliVue MX800 (Philips, Eindhoven, The Netherlands). The catheter–manometer system was flushed with 0.9% saline to minimize air in the line from catheter to monitor before measurements would be used in the comparison with VCM pressures.

Continuous finger arterial and intra-arterial pressures were measured simultaneously. In the first minutes of measurement the positions of the finger cuff and pressure transducer were checked for possible hydrostatic level errors and, if necessary, readjusted. Subsequently, BP was measured for 15 min. During the BP measurement, no therapeutic changes were made. The non-invasive and invasive arterial blood pressure curves of all patients were A/D converted at 200 Hz and stored for off-line analysis [15–20].

### Study endpoints

The main study parameters were the differences in systolic, diastolic and mean BP between VCM and intra-arterial measurements. Additionally, beat-to-beat variability was quantified.

### Data analyses

Data of all patients were analyzed by comparing each single-beat BP curve of the VCM with the corresponding curve obtained from intra-arterial measurement. Data were discarded if the intra-arterial BP signal was visually damped or when the automatic calibration of the Nexfin (Physiocal^®^) occurred more often than once every 30 heartbeats. The VCM device automatically translates BP to corresponding brachial artery levels. Since systolic BP is physiologically amplified between the brachial and radial artery, comparing VCM directly to radial intra-arterial pressures would lead to underestimation. Therefore, the intra-arterial radial artery pressure was corrected for (age-adjusted) BP amplification to reconstruct brachial artery pressures (Beatscope 1.1, TNO-BMI, Amsterdam, The Netherlands).

**Fig. 1:**
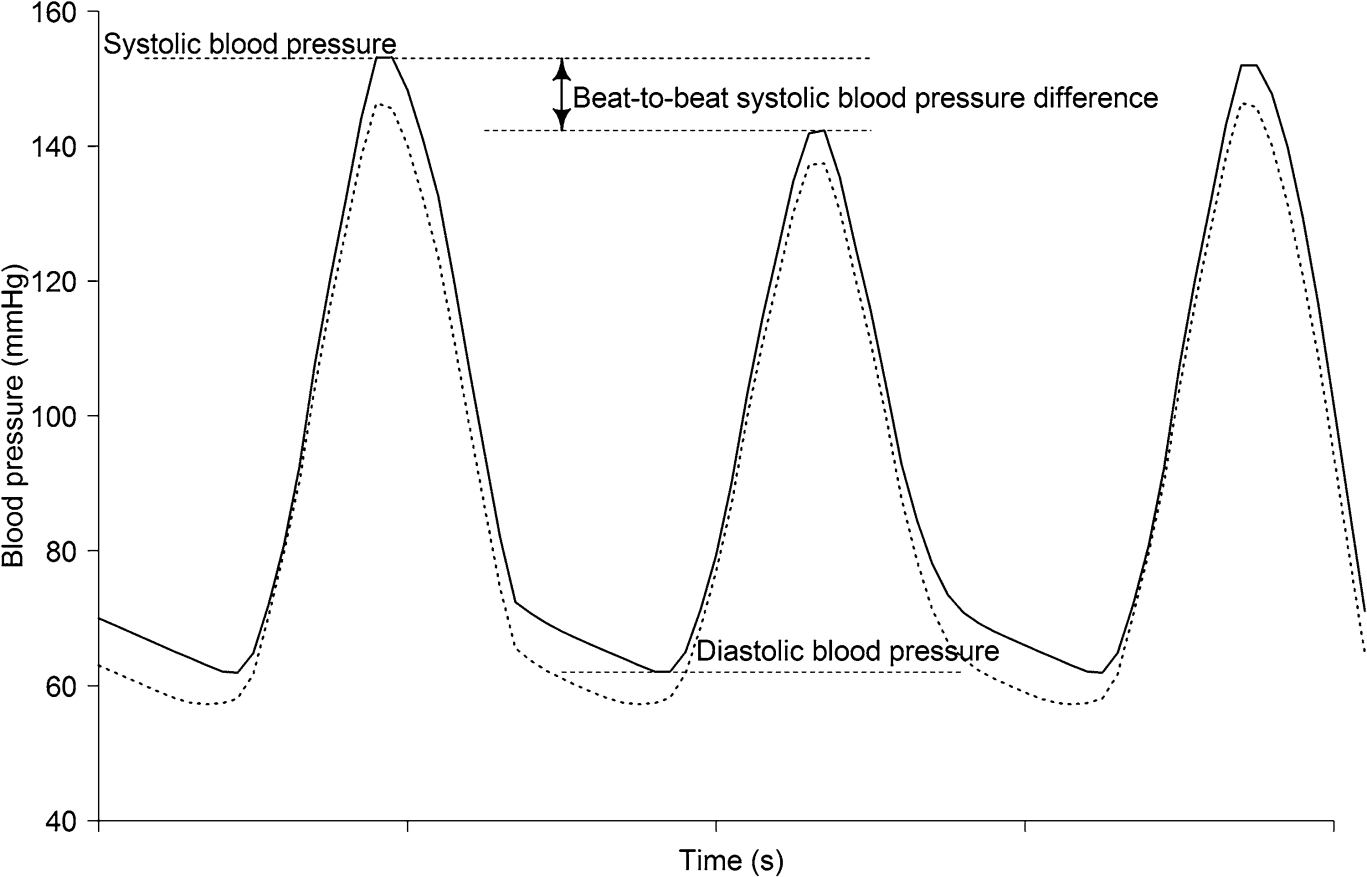
Measurement of blood pressure by VCM (*continues line*) and intra-arterial blood pressure (*dotted line*) representative for blood pressure comparison of all patients between the methods. *VCM* volume-clamp method

For each patient, the first 150 technically successful single-beat BPs (Physiocal >30) were selected. Data analyses (all data collectively) was performed using linear regression analysis, intra-class correlation and Bland Altman plotting for systolic, diastolic and mean BP. A mean difference of 5 (SD 8) mmHg (accuracy and precision) was considered acceptable as proposed by the Association for the Advancement of Medical Instrumentation American National Standard ANSI/AAMI/ISO 2009 guidelines [23]. However, the proposed “zero-zone method”, which is less suitable for fluctuating BPs or continuous measurements, was not applied [24]. Intra-individual beat-to-beat differences (absolute difference with the previous beat) of systolic BP (Fig. 1) of the VCM and intra-arterial measurements were compared to analyze whether the VCM device was able to accurately track beat-to-beat changes of systolic BP in patients with AF. We also performed a 4-quadrant plot analysis and concordance analysis on the percentage of change in systolic BP over time between VCM and intra-arterial measurements. An exclusion zone of 5% has been used, since intra-arterial systolic blood pressure measurements in patients with sinus rhythm showed variability between 5 and 10% [25]. The variability within this range is not solely due to the presence of atrial fibrillation but also due to spontaneous (e.g., respiration related) variability. Beat-to-beat fluctuations in diastolic BP were negligible compared to systolic fluctuations.

Data analyses of all single-beat BP measurements (more than first 150 technically successful measurements per patient) were similarly performed for each patient separately. The mean of the individual SDs was calculated as a measure of individual consistency (“Within-subject precision”) [18].

The statistical software package SPSS was used for statistical analyses (IBM Corp. Released 2011. IBM SPSS Statistics for Windows, Version 20.0. Armonk, NY: IBM Corp).

## Results

We included 31 patients with AF and 10 with SR between June 2013 and January 2014. Clinical characteristics are displayed in Table 1. Most patients were post-operative after cardiac surgery or carotid endarterectomy, others were recovering from sepsis or respiratory failure.

**Table 1:** Characteristics of the study population

	Atrial fibrillation (n = 31)	Sinus rhythm (n = 10)	p-value
Age (years)	74 (9)	64 (17)	0.01
Male sex	19 (61%)	6 (60%)	0.94
Heart rate (bpm)	94 (18)	81 (17)	0.06
Noradrenaline (µg/kg/min)	0.04 (0–0.20)	0.01 (0–0.05)	0.58
Systolic blood pressure (mmHg)	122 (22)	126 (24)	0.55
Diastolic blood pressure (mmHg)	64 (11)	63 (10)	0.78
Mean blood pressure (mmHg)	82 (14)	83 (14)	0.63

All data are displayed as mean ± SD or n (%), noradrenaline is displayed as mean (total range); blood pressure measurements are intra-arteria

*Bpm* beats per minute, *µg*/*kg*/*min* microgram per kilogram per minute

**Fig. 2:**
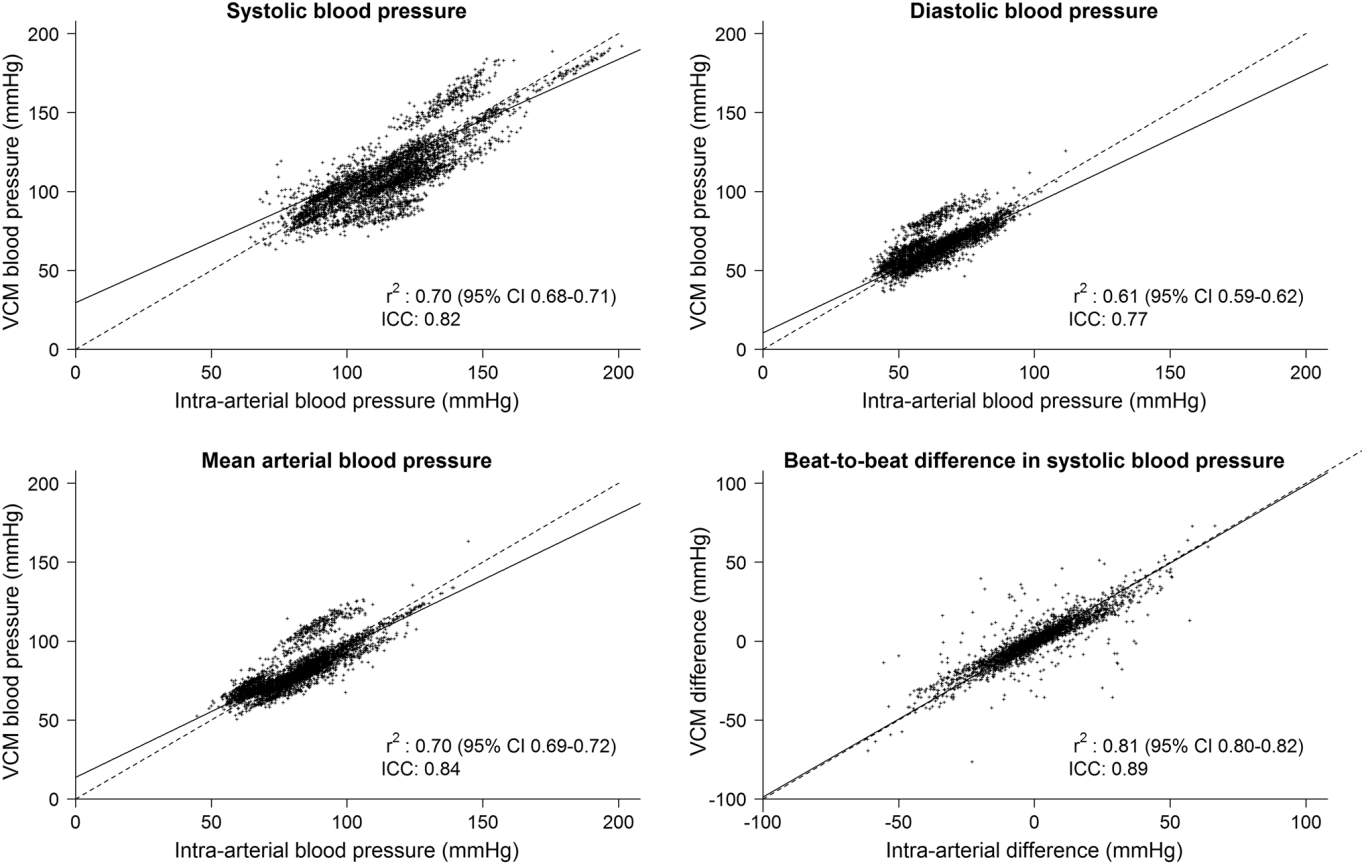
Regression analysis of VCM versus reconstructed arterial brachialis artery blood pressure in patients with atrial fibrillation. Each point represents a paired blood pressure measurement per beat. *VCM* volume-clamp method, *r* correlation coefficient, *CI* confidence interval, *ICC* intra-class correlation

Accuracy data are listed in Table 2. Overall (analyzing all data collectively), VCM underestimated systolic BP by a mean of 4 mmHg (SD 12) compared to intra-arterial measurement. For diastolic BP and mean BP, the results were comparable for both methods. The accuracy and precision of VCM in patients with AF corresponded to that in patients with SR. Absolute beat-to-beat differences of systolic BP were also accurately measured by VCM compared to intra-arterial measurements, indicating that pulse inequality can be accurately tracked by VCM. Intra-class correlations were high for both AF and SR patients. Correlation plots are presented for AF in Fig. 2.

**Table 2:** VCM versus intra-arterial blood pressure measurements

	VCM (mmHg)	Intra-arterial (mmHg)	Mean difference (mmHg)	Limits of agreement (bias ± 2 SD; mmHg)	Percentage error (%)	r² (p-value)	ICC
Atrial fibrillation (n = 31)							
Systolic blood pressure	111 (22)	115 (20)	−4 (12)	−28 to 20	21	0.70 (<0.01)	0.82
Diastolic blood pressure	65 (10)	64 (11)	1 (7)	−13 to 15	22	0.61 (<0.01)	0.77
Mean blood pressure	81 (13)	81 (13)	0 (8)	−15 to 15	18	0.70 (<0.01)	0.84
Absolute beat-to-beat systolic blood pressure difference	4.5 (1.8–10.5)	5.0 (2.0–11.3)	1.5 (0.5–3.8)	−11 to 11	NA	0.81 (<0.01)	0.89
Sinus rhythm (n = 10)							
Systolic blood pressure	110 (22)	116 (22)	−6 (10)	−26 to 15	18	0.78 (<0.01)	0.85
Diastolic blood pressure	67 (9)	62 (10)	4 (6)	−7 to 16	19	0.67 (<0.01)	0.73
Mean blood pressure	82 (12)	81 (14)	1 (5)	−10 to 11	13	0.87 (<0.01)	0.92
Absolute beat-to-beat systolic blood pressure difference	2.0 (0.75–4.3)	2.5 (1.0–5.3)	1.0 (0.5–1.8)	−4 to 7	NA	0.71 (<0.01)	0.82

All data are displayed as mean (SD) or median (interquartile range)

*VCM* volume-clamp method, *SD* standard deviation, *r* correlation coefficient, *ICC* intra-class correlation

**Fig. 3:**
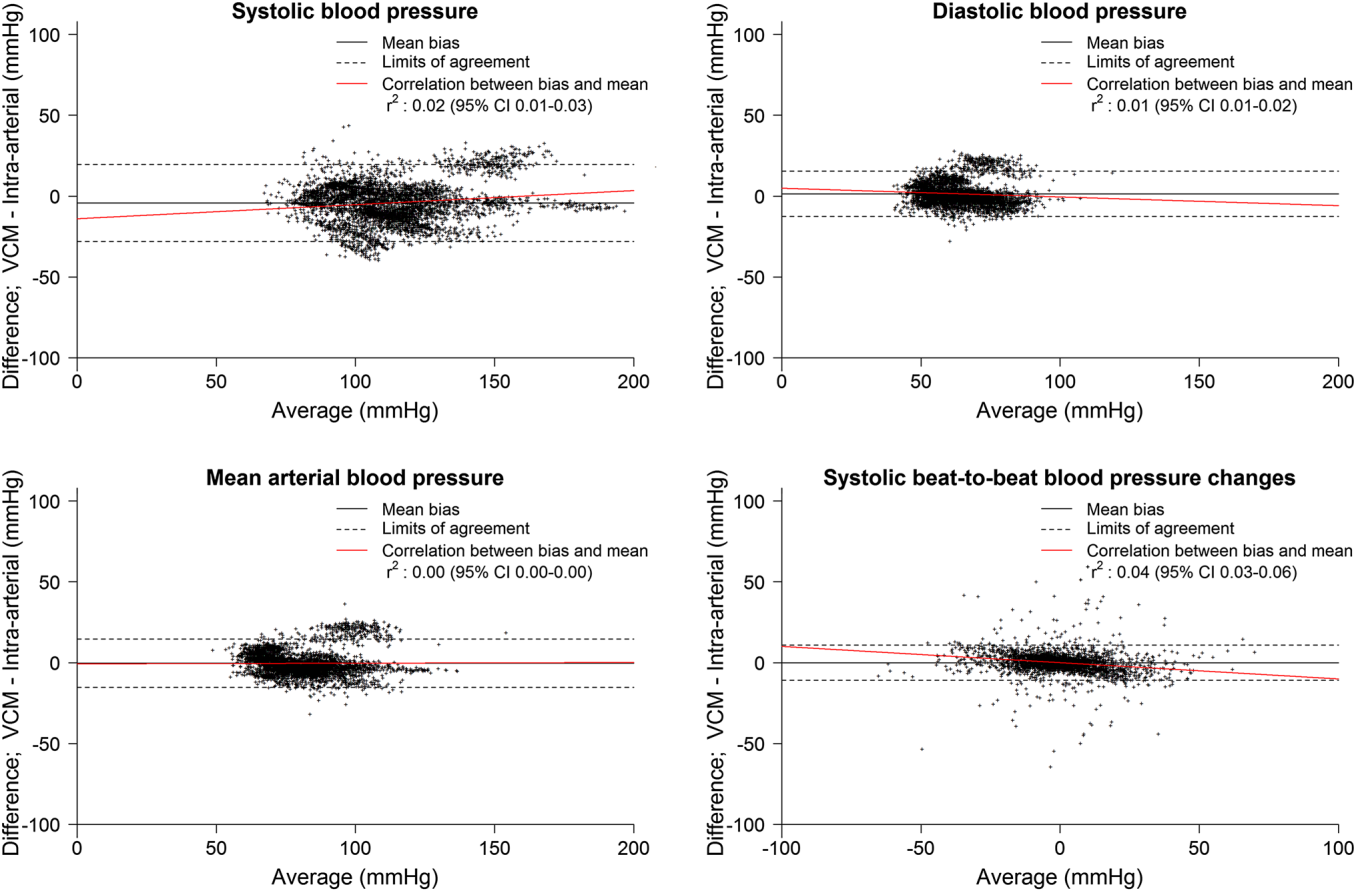
Bland Altman plots of blood pressure measurements per beat; VCM versus reconstructed brachial artery blood pressure in patients with atrial fibrillation. *VCM* volume-clamp method, *r* correlation coefficient, *CI* Confidence interval

**Fig. 4:**
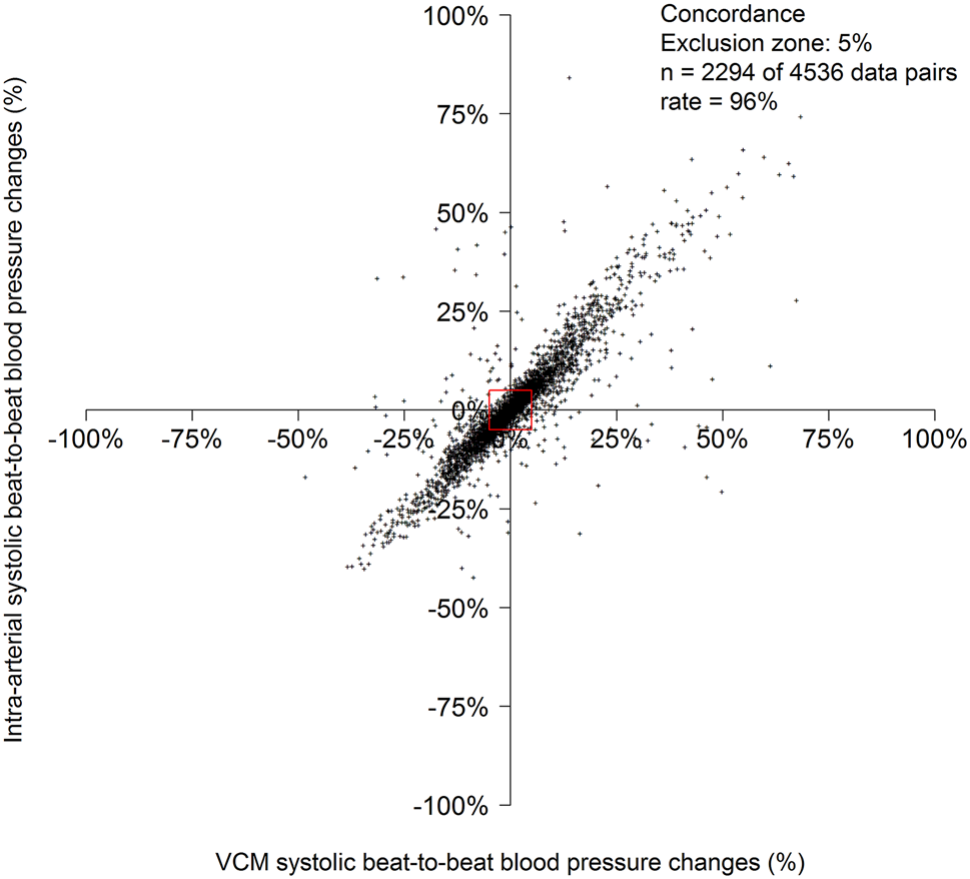
4-quadrant plot analyses of the percentage beat-to-beat blood pressure change of VCM versus intra-arterial measurements. Each point represents a paired blood pressure measurement per beat. *VCM* volume-clamp method

Bland Altman plots and analyses for systolic, diastolic and mean BP, as well as for beat-to-beat difference are displayed in Fig. 3 and Table 2. 4-quadrant plot analysis for beat-to-beat systolic blood pressure differences with an exclusion zone of 5% showed a concordance rate of 96% (Fig. 4).

Data analyses of data on patient level are listed in supplemental Table 1. Within-subject precision of systolic BP was accurate with a mean SD of 5.1 (SD 2.6). As expected, within-subject precision for systolic BP was better than group level precision (SD 12).

Notably, in one-third of the patients (10/31 patients) with AF and SR (3/10 patients; supplemental Table 2) there was a substantial difference of >10 mmHg between VCM and intra-arterial systolic BP measurements. In these 13 patients, the VCM was not accurate and precise compared to the intra-arterial BP. The most plausible explanation in all patients with AF and SR can be found in their baseline characteristics. Most patients used low-dose noradrenaline and/or had somewhat pale and cold hands or minor edema, but none severe enough for exclusion (supplemental Table 3). A regression model between the bias of systolic BP measurements (VCM minus intra-arterial measurements) and low-dose noradrenaline, minor edema, and minor paleness and cold hands, showed no significant relation between the bias and low-dose noradrenaline or pale cold hands (p-values: 0.66 and 0.26 respectively), however, there was a significant relation between bias and minor edema (p-value: 0.04) with an estimated 7.7 mmHg lower measurement with VCM compared to intra-arterial BP measurements. Absolute beat-to-beat differences of systolic BP were accurate (median absolute beat-to-beat difference <4.0 mmHg) in virtually all patients with AF except for patient 5.

Analyses of data that are first aggregated to individual patient level (average values of patients mean differences and ICC; supplemental Table 4) yield virtually equal results compared to collective data analyses (all single-beat BP analyzed together, without prior aggregation to patient level).

## Discussion

The most important finding in this study is that the non-invasive VCM method is accurate in measuring beat-to-beat BP in patients with AF. The VCM method could thus provide an easy, reliable and accessible method for clinical use, as well as for epidemiological studies of BP in AF, and validation studies for other BP monitoring methods and systems in AF.

Mean values of systolic BP measurements were accurate with the VCM method (<5 mmHg), but the standard deviation for systolic BP (12 mmHg) is above the limit proposed by the Association for the Advancement of Medical Instrumentation American National Standard ANSI/AAMI/ISO 2009 guidelines (mean difference of maximum 5 mmHg; SD 8 mmHg) [23]. For diastolic and mean BP measurements, the VCM does meet the ANSI/AAMI/ISO criteria.

However, whether this standard benchmark for validation is appropriate for AF is questionable, since a “zero-zone method” [25] is proposed in the AAMI guidelines [23, 24], which is less suitable for fluctuating BPs or continuous measurements. Applying a zero-zone method instead of a direct comparison of methods to such measurements attenuates mean differences and standard deviations, causing bias of the accuracy data towards meeting the AAMI criteria [24].

Our results indicate that the VCM method is appropriate for the validation of other BP measurements methods and devices in patients with AF. Although VCM is currently recommended for non-invasive hemodynamic monitoring in some critical ill patients, these studies did not investigate the validity in patients with AF solely [26]. As mentioned, in patients with AF, the beat-to-beat variation in blood pressure is substantially larger than in patients with SR. Therefore, validation studies in patients with AF are of major importance to provide tools for blood pressure monitoring not only in postoperative patients. Our study specifically addresses these patients in order to allow for extrapolation of our findings to even less ill AF patients, for example in the clinical ward or outpatient clinic. Such studies have rarely been performed. One example is a recent study of 135 patients with arrhythmia (including 88% of the patients with AF) and an intra-arterial line, in which measurement of arterial BP with an oscillometric device fulfilled the ISO criteria for mean and diastolic BP. Systolic BP was accurate, but not precise for both patients with arrhythmia or regular rhythm (mean bias −1.6 mmHg; SD 10.4 mmHg) [27]. It is important to stress, however, that no oscillometric device is the same in term of device technology and computational algorithm, and separate validation in AF is thus required for each system. Finally, oscillometric devices or in fact any other method not registering beat-to-beat BP cannot address BP fluctuations between heart beats, which may confer clinically relevant information [28, 29].

Some limitations of this study should be considered. First, this is a validation study in patients that were hospitalized and either post-operative or critically ill patients. However, since intra-arterial measurements in stable outpatients are ethically problematic, this type of study may simply be unfeasible in outpatients.

In addition, BP measurement with VCM was inaccurate in one-third of all patients. However, results where similar for patient with SR. In most cases where VCM differed substantially from intra-arterial measurement, vasopressors and/or peripheral edema provided a plausible explanation for this. Also, the small within-subject variability indicates that the differences remain stable. It has been described previously that in patients with critical illness the signal of the systolic BP is damped [30]. It thus seems prudent to trust the VCM method only in patients with adequate peripheral perfusion in non-edematous fingers.

Finally, the validation study required adjustment of radial intra-arterial BP values to corresponding brachial levels, which were the basis of the original VCM validation studies. Intra-arterial measurements of BP in the brachial artery could be more accurate as a reference method.

In conclusion, VCM can measure BP, including its beat-to-beat fluctuations, accurately in patients with AF. This result enables a range of future studies addressing both measurement techniques as well as the clinical importance of BP (fluctuations) in patients with AF.

## Electronic supplementary material

Below is the link to the electronic supplementary material.

Supplementary material 1 (DOCX 28 KB)

Supplementary material 2 (DOCX 19 KB)

Supplementary material 3 (DOCX 18 KB)

Supplementary material 4 (DOCX 17 KB)

## References

[1] Go AS, Hylek EM, Phillips KA, Chang Y, Henault LE, Selby JV, Singer DE (2001). Prevalence of diagnosed atrial fibrillation in adults: national implications for rhythm management and stroke prevention: the AnTicoagulation and Risk Factors in Atrial Fibrillation (ATRIA) Study. Jama.

[2] Mozaffarian D, Benjamin EJ, Go AS, Arnett DK, Blaha MJ, Cushman M, de Ferranti S, Despres JP, Fullerton HJ, Howard VJ, Huffman MD, Judd SE, Kissela BM, Lackland DT, Lichtman JH, Lisabeth LD, Liu S, Mackey RH, Matchar DB, McGuire DK, Mohler ER, Moy CS, Muntner P, Mussolino ME, Nasir K, Neumar RW, Nichol G, Palaniappan L, Pandey DK, Reeves MJ, Rodriguez CJ, Sorlie PD, Stein J, Towfighi A, Turan TN, Virani SS, Willey JZ, Woo D, Yeh RW, Turner MB, American Heart Association Statistics C, Stroke Statistics S (2015). Heart disease and stroke statistics–2015 update: a report from the American Heart Association. Circulation.

[3] Lim SS, Vos T, Flaxman AD, Danaei G, Shibuya K, Adair-Rohani H, Amann M, Anderson HR, Andrews KG, Aryee M, Atkinson C, Bacchus LJ, Bahalim AN, Balakrishnan K, Balmes J, Barker-Collo S, Baxter A, Bell ML, Blore JD, Blyth F, Bonner C, Borges G, Bourne R, Boussinesq M, Brauer M, Brooks P, Bruce NG, Brunekreef B, Bryan-Hancock C, Bucello C, Buchbinder R, Bull F, Burnett RT, Byers TE, Calabria B, Carapetis J, Carnahan E, Chafe Z, Charlson F, Chen H, Chen JS, Cheng AT, Child JC, Cohen A, Colson KE, Cowie BC, Darby S, Darling S, Davis A, Degenhardt L, Dentener F, Des Jarlais DC, Devries K, Dherani M, Ding EL, Dorsey ER, Driscoll T, Edmond K, Ali SE, Engell RE, Erwin PJ, Fahimi S, Falder G, Farzadfar F, Ferrari A, Finucane MM, Flaxman S, Fowkes FG, Freedman G, Freeman MK, Gakidou E, Ghosh S, Giovannucci E, Gmel G, Graham K, Grainger R, Grant B, Gunnell D, Gutierrez HR, Hall W, Hoek HW, Hogan A, Hosgood HD, Hoy D, Hu H, Hubbell BJ, Hutchings SJ, Ibeanusi SE, Jacklyn GL, Jasrasaria R, Jonas JB, Kan H, Kanis JA, Kassebaum N, Kawakami N, Khang YH, Khatibzadeh S, Khoo JP, Kok C, Laden F, Lalloo R, Lan Q, Lathlean T, Leasher JL, Leigh J, Li Y, Lin JK, Lipshultz SE, London S, Lozano R, Lu Y, Mak J, Malekzadeh R, Mallinger L, Marcenes W, March L, Marks R, Martin R, McGale P, McGrath J, Mehta S, Mensah GA, Merriman TR, Micha R, Michaud C, Mishra V, Mohd Hanafiah K, Mokdad AA, Morawska L, Mozaffarian D, Murphy T, Naghavi M, Neal B, Nelson PK, Nolla JM, Norman R, Olives C, Omer SB, Orchard J, Osborne R, Ostro B, Page A, Pandey KD, Parry CD, Passmore E, Patra J, Pearce N, Pelizzari PM, Petzold M, Phillips MR, Pope D, Pope CA, Powles J, Rao M, Razavi H, Rehfuess EA, Rehm JT, Ritz B, Rivara FP, Roberts T, Robinson C, Rodriguez-Portales JA, Romieu I, Room R, Rosenfeld LC, Roy A, Rushton L, Salomon JA, Sampson U, Sanchez-Riera L, Sanman E, Sapkota A, Seedat S, Shi P, Shield K, Shivakoti R, Singh GM, Sleet DA, Smith E, Smith KR, Stapelberg NJ, Steenland K, Stockl H, Stovner LJ, Straif K, Straney L, Thurston GD, Tran JH, Van Dingenen R, van Donkelaar A, Veerman JL, Vijayakumar L, Weintraub R, Weissman MM, White RA, Whiteford H, Wiersma ST, Wilkinson JD, Williams HC, Williams W, Wilson N, Woolf AD, Yip P, Zielinski JM, Lopez AD, Murray CJ, Ezzati M, AlMazroa MA, Memish ZA (2012). A comparative risk assessment of burden of disease and injury attributable to 67 risk factors and risk factor clusters in 21 regions, 1990–2010: a systematic analysis for the Global Burden of Disease Study 2010. The Lancet.

[4] Schnabel RB, Sullivan LM, Levy D, Pencina MJ, Massaro JM, D’Agostino RB, Newton-Cheh C, Yamamoto JF, Magnani JW, Tadros TM, Kannel WB, Wang TJ, Ellinor PT, Wolf PA, Vasan RS, Benjamin EJ (2009). Development of a risk score for atrial fibrillation (Framingham Heart Study): a community-based cohort study. The Lancet.

[5] Singer DE, Chang Y, Borowsky LH, Fang MC, Pomernacki NK, Udaltsova N, Reynolds K, Go AS (2013). A new risk scheme to predict ischemic stroke and other thromboembolism in atrial fibrillation: the ATRIA study stroke risk score. J Am Heart Assoc.

[6] Lip GY, Halperin JL (2010). Improving stroke risk stratification in atrial fibrillation. Am J Med.

[7] Pisters R, Lane DA, Nieuwlaat R, de Vos CB, Crijns HJ, Lip GY (2010). A novel user-friendly score (HAS-BLED) to assess 1-year risk of major bleeding in patients with atrial fibrillation: the Euro Heart Survey. Chest.

[8] Sykes D, Dewar R, Mohanaruban K, Donovan K, Nicklason F, Thomas DM, Fisher D (1990). Measuring blood pressure in the elderly: does atrial fibrillation increase observer variability?. BMJ.

[9] January CT, Wann LS, Alpert JS, Calkins H, Cigarroa JE, Cleveland JC, Conti JB, Ellinor PT, Ezekowitz MD, Field ME, Murray KT, Sacco RL, Stevenson WG, Tchou PJ, Tracy CM, Yancy CW, Members AATF (2014). 2014 AHA/ACC/HRS guideline for the management of patients with atrial fibrillation: a report of the American College of Cardiology/American Heart Association Task Force on practice guidelines and the Heart Rhythm Society. Circulation.

[10] Camm AJ, Lip GY, De Caterina R, Savelieva I, Atar D, Hohnloser SH, Hindricks G, Kirchhof P, ESCCfP Guidelines (2012). 2012 focused update of the ESC Guidelines for the management of atrial fibrillation: an update of the 2010 ESC Guidelines for the management of atrial fibrillation. Developed with the special contribution of the European Heart Rhythm Association. Eur Heart J.

[11] Kaliujnaya VS, Kalyuzhny SI (2005). The assessment of blood pressure in atrial fibrillation. Comput Cardiol.

[12] Pagonas N, Schmidt S, Eysel J, Compton F, Hoffmann C, Seibert F, Hilpert J, Tschope C, Zidek W, Westhoff TH (2013). Impact of atrial fibrillation on the accuracy of oscillometric blood pressure monitoring. Hypertension.

[13] Stergiou GS, Kollias A, Destounis A, Tzamouranis D (2012). Automated blood pressure measurement in atrial fibrillation: a systematic review and meta-analysis. J Hypertens.

[14] Bogert LW, Wesseling KH, Schraa O, Van Lieshout EJ, de Mol BA, van Goudoever J, Westerhof BE, van Lieshout JJ (2010). Pulse contour cardiac output derived from non-invasive arterial pressure in cardiovascular disease. Anaesthesia.

[15] Eeftinck Schattenkerk DW, van Lieshout JJ, van den Meiracker AH, Wesseling KR, Blanc S, Wieling W, van Montfrans GA, Settels JJ, Wesseling KH, Westerhof BE (2009). Nexfin noninvasive continuous blood pressure validated against Riva-Rocci/Korotkoff. Am J Hypertens.

[16] Fischer MO, Avram R, Carjaliu I, Massetti M, Gerard JL, Hanouz JL, Fellahi JL (2012). Non-invasive continuous arterial pressure and cardiac index monitoring with Nexfin after cardiac surgery. Br J Anaesth.

[17] Garnier RP, van der Spoel AG, Sibarani-Ponsen R, Markhorst DG, Boer C (2012). Level of agreement between Nexfin non-invasive arterial pressure with invasive arterial pressure measurements in children. Br J Anaesth.

[18] Martina JR, Westerhof BE, van Goudoever J, de Beaumont EM, Truijen J, Kim YS, Immink RV, Jobsis DA, Hollmann MW, Lahpor JR, de Mol BA, van Lieshout JJ (2012). Noninvasive continuous arterial blood pressure monitoring with Nexfin(R). Anesthesiology.

[19] Sipkens LM, Treskes K, Ariese-Beldman K, Veerman DP, Boer C (2011). Application of Nexfin noninvasive beat-to-beat arterial blood pressure monitoring in autonomic function testing. Blood Press Monit.

[20] van der Spoel AG, Voogel AJ, Folkers A, Boer C, Bouwman RA (2012). Comparison of noninvasive continuous arterial waveform analysis (Nexfin) with transthoracic doppler echocardiography for monitoring of cardiac output. J Clin Anesth.

[21] Chin KY, Panerai RB (2012). Comparative study of Finapres devices. Blood Press Monit.

[22] Imholz BP, Wieling W, van Montfrans GA, Wesseling KH (1998). Fifteen years experience with finger arterial pressure monitoring: assessment of the technology. Cardiovasc Res.

[23] Advancing Safety in Medical Technology (AAMI). ANSI/AAMI/ISO 81060-2:2013. Non-invasive sphygmomanometers—part 2: clinical investigation of automated measurement type. 2013. http://my.aami.org/aamiresources/previewfiles/8106002_1306_preview.pdf.

[24] Fortin J, Lerche K, Flot-Zinger D, O’Brien T (2015). Is the standard supplied by the association for the advancement of medical instrumentation the measure of all things for noninvasive continuous hemodynamic devices?. Anesthesiology.

[25] Mancia G, Ferrari A, Gregorini L, Parati G, Pomidossi G, Bertinieri G, Grassi G, di Rienzo M, Pedotti A, Zanchetti A (1983). Blood pressure and heart rate variabilities in normotensive and hypertensive human beings. Circ Res.

[26] Teboul JL, Saugel B, Cecconi M, De Backer D, Hofer CK, Monnet X, Perel A, Pinsky MR, Reuter DA, Rhodes A, Squara P, Vincent JL, Scheeren TW (2016). Less invasive hemodynamic monitoring in critically ill patients. Intensive Care Med.

[27] Lakhal K, Ehrmann S, Martin M, Faiz S, Reminiac F, Cinotti R, Capdevila X, Asehnoune K, Blanloeil Y, Rozec B, Boulain T (2015). Blood pressure monitoring during arrhythmia: agreement between automated brachial cuff and intra-arterial measurements. Br J Anaesth.

[28] Rothwell PM, Howard SC, Dolan E, O’Brien E, Dobson JE, Dahlof B, Poulter NR, Sever PS, Ascot B, Investigators MRCT (2010). Effects of beta blockers and calcium-channel blockers on within-individual variability in blood pressure and risk of stroke. Lancet Neurol.

[29] Webb AJ, Fischer U, Mehta Z, Rothwell PM (2010). Effects of antihypertensive-drug class on interindividual variation in blood pressure and risk of stroke: a systematic review and meta-analysis. The Lancet.

[30] Hohn A, Defosse JM, Becker S, Steffen C, Wappler F, Sakka SG (2013). Non-invasive continuous arterial pressure monitoring with Nexfin does not sufficiently replace invasive measurements in critically ill patients. Br J Anaesth.

