# Supplementary material for: Comparing volume-clamp method and intra-arterial blood pressure measurements in patients with atrial fibrillation admitted to the intensive or medium care unit
Source: J Clin Monit Comput. 2017 Jul 7;32(3):439–46. doi: 10.1007/s10877-017-0044-9 (PMC5943389; doi:10.1007/s10877-017-0044-9)
Supplement: Supplementary file 1 — Supplementary material 1 (DOCX 28 KB) [file 10877_2017_44_MOESM1_ESM.docx]

| **Supplemental table 1: Individual analyzed data** | | | | | | | | | | | | | | | | |
| --- | --- | --- | --- | --- | --- | --- | --- | --- | --- | --- | --- | --- | --- | --- | --- | --- |
| Patient | SBP VCM | SBP IA | SBP diff. | r² SBP | DBP VCM | DBP IA | DBP diff. | r² DBP | MAP VCM | MAP IA | MAP diff. | r² MAP | B-to-B VCM | B-to-B IA | Absolute B-to-B diff. | r²  B-to-B |
| 1 | 158 (9) | 136 (11) | 22 (4) | 0.88 | 86 (6) | 63 (6) | 23 (2) | 0.89 | 113 (7) | 89 (7) | 24 (2) | 0.90 | 7.5 (3.25-14.25) | 10.0 (4.50-18.25) | 2.8 (1.5-4.8) | 0.96 |
| 2 | 93 (7) | 96 (8) | –3 (4) | 0.72 | 55 (6) | 53 (6) | 1 (2) | 0.90 | 70 (6) | 69 (7) | 1 (3) | 0.86 | 2.3 (1.00-4.50) | 2.5 (1.00-4.75) | 1.0 (0.5-1.8) | 0.95 |
| 3 | 126 (12) | 125 (10) | 0 (3) | 0.93 | 60 (8) | 62 (7) | –2 (3) | 0.91 | 81 (9) | 82 (7) | –1 (3) | 0.94 | 6.3 (3.00-12.50) | 5.5 (2.50-11.25) | 1.0 (0.5-1.8) | 0.98 |
| 4 | 96 (12) | 95 (10) | 1 (10) | 0.31 | 68 (6) | 72 (11) | –4 (9) | 0.23 | 77 (7) | 80 (9) | –2 (8) | 0.25 | 6.5 (3.00-11.25) | 5.0 (2.00-9.50) | 3.5 (1.0-9.0) | 0.24 |
| 5 | 92 (12) | 89 (12) | 3 (14) | 0.09 | 55 (5) | 54 (6) | 1 (6) | 0.08 | 67 (6) | 66 (7) | 1 (7) | 0.19 | 12.0 (5.50-21.25) | 12.0 (5.75-20.25) | 14.3 (6.3-24.8) | 0.04 |
| 6 | 99 (6) | 91 (7) | 8 (3) | 0.81 | 54 (3) | 46 (3) | 8 (2) | 0.78 | 68 (4) | 60 (4) | 8 (1) | 0.87 | 3.0 (1.50-5.50) | 3.0 (1.50-5.25) | 0.5 (0.3-1.0) | 0.73 |
| 7 | 119 (7) | 124 (6) | –5 (9) | 0.00* | 63 (5) | 66 (5) | –3 (8) | 0.00* | 84 (6) | 89 (5) | –5 (8) | 0.00* | 2.1 (1.00-3.50) | 2.3 (1.00-3.75) | 2.8 (1.0-5.0) | 0.00* |
| 8 | 124 (6) | 120 (5) | 4 (2) | 0.84 | 68 (5) | 66 (6) | 2 (2) | 0.90 | 87 (5) | 84 (5) | 3 (2) | 0.88 | 4.5 (2.00-7.50) | 3.8 (1.75-6.50) | 0.8 (0.5-1.5) | 0.96 |
| 9 | 111 (14) | 100 (14) | 10 (7) | 0.73 | 61 (7) | 49 (6) | 11 (3) | 0.87 | 73 (7) | 61 (6) | 12 (3) | 0.75 | 10.0 (4.75-18.75) | 12.0 (5.75-21.75) | 2.3 (1.0-4.3) | 0.81 |
| 10 | 89 (5) | 111 (9) | –22 (6) | 0.51 | 64 (5) | 53 (7) | 11 (3) | 0.77 | 75 (4) | 78 (5) | –3 (5) | 0.31 | 2.3 (1.13-4.38) | 5.0 (2.50-9.63) | 2.8 (1.5-5.3) | 0.67 |
| 11 | 84 (9) | 103 (11) | –19 (4) | 0.85 | 52 (4) | 55 (4) | –4 (2) | 0.82 | 65 (6) | 73 (6) | –8 (2) | 0.85 | 6.0 (3.00-11.00) | 9.8 (4.50-17.25) | 3.8 (1.5-6.5) | 0.98 |
| 12 | 134 (15) | 130 (11) | 4 (9) | 0.62 | 73 (8) | 71 (7) | 2 (4) | 0.74 | 96 (10) | 94 (8) | 2 (5) | 0.71 | 7.8 (3.75-14.00) | 8.3 (4.00-14.00) | 1.3 (0.5-2.5) | 0.94 |
| 13 | 102 (7) | 110 (8) | –8 (5) | 0.66 | 60 (6) | 60 (6) | 0 (2) | 0.85 | 76 (6) | 78 (6) | –2 (2) | 0.84 | 5.0 (2.50-8.25) | 5.3 (2.75-8.50) | 1.3 (0.5-2.8) | 0.71 |
| 14 | 126 (12) | 146 (13) | –20 (5) | 0.83 | 80 (7) | 83 (7) | –3 (5) | 0.50 | 99 (8) | 107 (10) | –9 (5) | 0.73 | 8.3 (3.75-13.50) | 10.5 (5.00-17.75) | 2.8 (1.3-4.8) | 0.97 |
| 15 | 83 (9) | 85 (9) | –2 (4) | 0.84 | 58 (5) | 52 (6) | 6 (2) | 0.83 | 67 (6) | 64 (6) | 3 (2) | 0.90 | 3.0 (1.50-5.25) | 4.0 (1.75-6.63) | 1.0 (0.5-2.0) | 0.89 |
| 16 | 101 (6) | 96 (6) | 5 (3) | 0.77 | 52 (3) | 50 (3) | 3 (2) | 0.53 | 65 (4) | 61 (3) | 4 (2) | 0.57 | 2.0 (1.00-3.50) | 2.3 (1.00-3.75) | 0.5 (0.3-0.8) | 0.95 |
| 17 | 109 (8) | 112 (7) | –3 (3) | 0.82 | 66 (5) | 65 (5) | 1 (2) | 0.82 | 81 (6) | 82 (6) | –1 (2) | 0.89 | 4.0 (1.75-6.25) | 3.8 (2.00-6.00) | 1.0 (0.5-1.5) | 0.88 |
| 18 | 137 (12) | 138 (13) | –1 (4) | 0.90 | 77 (7) | 74 (8) | 4 (3) | 0.82 | 97 (8) | 95 (8) | 2 (3) | 0.84 | 10.5 (5.00-21.25) | 11.3 (5.25-22.50) | 1.3 (0.5-2.5) | 0.99 |
| 19 | 111 (11) | 113 (12) | –2 (5) | 0.84 | 74 (7) | 70 (7) | 4 (2) | 0.90 | 87 (8) | 85 (8) | 3 (3) | 0.89 | 9.0 (4.00-16.50) | 11.8 (5.25-20.25) | 2.5 (1.3-4.3) | 0.98 |
| 20 | 103 (12) | 112 (14) | –9 (5) | 0.87 | 62 (7) | 62 (8) | 0 (3) | 0.88 | 79 (8) | 81 (9) | –2 (3) | 0.86 | 6.5 (2.75-16.25) | 7.0 (2.75-21.25) | 2.5 (1.3-5.3) | 0.93 |
| 21 | 117 (14) | 121 (14) | -4 (4) | 0.91 | 68 (6) | 68 (7) | 0 (2) | 0.93 | 85 (8) | 87 (8) | –2 (2) | 0.93 | 11.0 (5.00-21.75) | 12.8 (5.75-23.00) | 1.8 (0.8-3.3) | 0.98 |
| 22 | 122 (10) | 124 (10) | –2 (4) | 0.84 | 79 (6) | 80 (7) | –1 (3) | 0.87 | 95 (7) | 96 (7) | –1 (3) | 0.85 | 8.8 (4.25-14.25) | 8.3 (3.50-13.19) | 1.5 (0.8-2.5) | 0.96 |
| 23 | 105 (7) | 103 (9) | 2 (7) | 0.47 | 69 (4) | 70 (8) | –1 (7) | 0.23 | 82 (5) | 82 (7) | –1 (6) | 0.28 | 6.0 (2.75-10.75) | 4.8 (2.00-8.75) | 2.3 (1.0-4.0) | 0.40 |
| 24 | 138 (18) | 122 (14) | 17 (5) | 0.94 | 75 (8) | 59 (8) | 16 (2) | 0.93 | 95 (11) | 80 (10) | 15 (3) | 0.95 | 8.5 (3.50-16.75) | 7.3 (3.25-15.00) | 1.8 (0.8-2.8) | 0.98 |
| 25 | 89 (5) | 118 (6) | –28 (5) | 0.43 | 66 (3) | 56 (5) | 11 (3) | 0.42 | 75 (4) | 75 (5) | 0 (4) | 0.38 | 2.3 (1.00-4.25) | 5.3 (2.25-9.25) | 3.3 (1.8-5.5) | 0.83 |
| 26 | 110 (7) | 121 (7) | –12 (2) | 0.94 | 67 (5) | 69 (5) | –2 (2) | 0.87 | 81 (5) | 87 (5) | –6 (1) | 0.92 | 2.8 (1.25-4.50) | 2.8 (1.25-4.50) | 0.5 (0.3-1.0) | 0.96 |
| 27 | 103 (13) | 119 (15) | –16 (6) | 0.86 | 61 (7) | 66 (8) | –4 (3) | 0.87 | 75 (7) | 82 (8) | –7 (3) | 0.86 | 13.0 (6.00-22.00) | 16.8 (7.50-27.75) | 3.8 (1.8-6.0) | 0.92 |
| 28 | 106 (9) | 127 (6) | –21 (6) | 0.54 | 54 (6) | 56 (5) | –2 (3) | 0.76 | 75 (6) | 82 (6) | –7 (4) | 0.57 | 1.5 (0.75-3.00) | 3.1 (1.50-6.06) | 1.6 (0.8-3.8) | 0.88 |
| 29 | 105 (9) | 99 (7) | 6 (3) | 0.90 | 65 (6) | 59 (6) | 5 (3) | 0.75 | 77 (6) | 71 (5) | 5 (3) | 0.79 | 3.3 (1.50-6.75) | 2.8 (1.25-5.75) | 1.0 (0.5-1.5) | 0.95 |
| 30 | 84 (4) | 85 (3) | –1 (3) | 0.53 | 61 (3) | 62 (4) | –2 (2) | 0.73 | 70 (3) | 72 (3) | –2 (2) | 0.58 | 1.3 (0.50-2.00) | 1.5 (0.50-2.50) | 0.5 (0.3-0.8) | 0.89 |
| 31 | 166 (10) | 162 (10) | 4 (5) | 0.77 | 76 (8) | 78 (8) | –2 (2) | 0.94 | 106 (9) | 107 (9) | –1 (2) | 0.94 | 3.3 (1.50-6.00) | 3.3 (1.50-6.00) | 0.8 (0.5-1.5) | 0.93 |
| VCM: Volume clamp method; IA: Intra-arterial; SBP: Systolic blood pressure; DBP: Diastolic blood pressure; MAP: Mean arterial pressure; B-to-B: Beat-to-beat blood pressure; diff.: Difference; r: Correlation coefficient. *p-value >0.05, all other r² are significant with p-value <0.01 | | | | | | | | | | | | | | | | |
