# Supplementary material for: Comparing volume-clamp method and intra-arterial blood pressure measurements in patients with atrial fibrillation admitted to the intensive or medium care unit
Source: J Clin Monit Comput. 2017 Jul 7;32(3):439–46. doi: 10.1007/s10877-017-0044-9 (PMC5943389; doi:10.1007/s10877-017-0044-9)
Supplement: Supplementary file 2 — Supplementary material 2 (DOCX 19 KB) [file 10877_2017_44_MOESM2_ESM.docx]

| **Supplemental table 2: Individual analyzed data for sinus rhythm** | | | | | | | | | | | | | | | | |
| --- | --- | --- | --- | --- | --- | --- | --- | --- | --- | --- | --- | --- | --- | --- | --- | --- |
| Patient | SBP VCM | SBP IA | SBP diff. | r² SBP | DBP VCM | DBP IA | DBP diff. | r² DBP | MAP VCM | MAP IA | MAP diff. | r² MAP | B-to-B VCM | B-to-B IA | Absolute B-to-B diff. | r² B-to-B |
| 1 | 158 (8) | 153 (6) | 5 (8) | 0.18 | 81 (6) | 81 (6) | 0 (7) | 0.06 | 109 (6) | 108 (5) | 1 (7) | 0.11 | 1.8 (0.75-2.75) | 2.5 (1.25-3.50) | 2.0 (1.0-4.0) | 0.03 |
| 2 | 139 (6) | 138 (7) | 0 (6) | 0.38 | 79 (3) | 72 (4) | 6 (2) | 0.68 | 97 (4) | 96 (5) | 1 (3) | 0.59 | 1.0 (0.50-1.75) | 1.5 (0.75-2.75) | 0.8 (0.3-1.5) | 0.53 |
| 3 | 91 (2) | 89 (3) | 2 (3) | 0.24 | 62 (1) | 50 (1) | 12 (1) | 0.23 | 73 (2) | 64 (2) | 9 (2) | 0.32 | 0.8 (0.25-1.50) | 1.0 (0.50-2.00) | 0.5 (0.3-0.8) | 0.89 |
| 4 | 105 (11) | 118 (12) | –13 (7) | 0.62 | 63 (5) | 68 (6) | –5 (4) | 0.56 | 79 (7) | 87 (8) | –8 (5) | 0.64 | 6.3 (3.75-9.25) | 6.8 (3.75-10.00) | 0.8 (0.3-1.5) | 0.54 |
| 5 | 114 (3) | 117 (3) | –3 (1) | 0.86 | 61 (3) | 62 (2) | –1 (1) | 0.83 | 80 (3) | 81 (3) | –1 (1) | 0.88 | 1.8 (1.00-2.75) | 1.8 (0.75-2.75) | 0.3 (0.3-0.5) | 0.93 |
| 6 | 96 (9) | 91 (8) | 5 (2) | 0.95 | 66 (5) | 59 (5) | 7 (1) | 0.96 | 76 (6) | 70 (6) | 6 (1) | 0.97 | 2.5 (1.25-4.25) | 3.8 (2.00-5.75) | 1.3 (0.5-2.0) | 0.95 |
| 7 | 112 (7) | 103 (12) | 8 (12) | 0.06 | 64 (4) | 60 (6) | 4 (7) | 0.01* | 80 (5) | 75 (6) | 5 (7) | 0.03 | 6.0 (2.00-8.75) | 5.3 (1.50-9.75) | 6.0 (2.0-10.9) | 0.02 |
| 8 | 111 (6) | 116 (5) | –5 (4) | 0.64 | 71 (4) | 67 (3) | 3 (2) | 0.67 | 86 (4) | 86 (4) | 0 (3) | 0.62 | 4.3 (2.00-6.50) | 4.5 (2.25-7.50) | 1.0 (0.5-1.5) | 0.96 |
| 9 | 129 (8) | 144 (9) | –15 (4) | 0.85 | 80 (5) | 72 (8) | 8 (4) | 0.88 | 97 (6) | 96 (8) | 1 (3) | 0.90 | 0.8 (0.25-1.25) | 1.0 (0.50-1.75) | 1.0 (0.5-1.8) | 0.66 |
| 10 | 85 (6) | 116 (8) | –30 (5) | 0.59 | 53 (5) | 46 (4) | 6 (5) | 0.26 | 65 (5) | 68 (5) | –3 (4) | 0.44 | 5.5 (2.50-8.00) | 8.8 (3.50-13.25) | 3.0 (1.3-5.1) | 0.92 |
| VCM: Volume clamp method; IA: Intra-arterial; SBP: Systolic blood pressure; DBP: Diastolic blood pressure; MAP: Mean arterial pressure; B-to-B: Beat-to-beat blood pressure; diff.: Difference; r: Correlation coefficient. *p-value =0.05, all other r² are significant with p-value <0.01 | | | | | | | | | | | | | | | | |
