# Supplementary material for: Comparing volume-clamp method and intra-arterial blood pressure measurements in patients with atrial fibrillation admitted to the intensive or medium care unit
Source: J Clin Monit Comput. 2017 Jul 7;32(3):439–46. doi: 10.1007/s10877-017-0044-9 (PMC5943389; doi:10.1007/s10877-017-0044-9)
Supplement: Supplementary file 3 — Supplementary material 3 (DOCX 18 KB) [file 10877_2017_44_MOESM3_ESM.docx]

| **Supplemental table 3: Baseline measurements of individual patients** | | | | |
| --- | --- | --- | --- | --- |
| Patient with AF | Gender | Noradrenaline (µg/kg/min) | Trace of edema (Y/N) | Pale/cold hands (Y/N) |
| 1 | F | 0 | N | N |
| 2 | M | 0.07 | N | N |
| 3 | M | 0.005 | Y | N |
| 4 | M | 0 | N | N |
| 5 | M | 0.10 | N | N |
| 6 | M | 0 | N | N |
| 7 | F | 0 | N | N |
| 8 | F | 0 | N | N |
| 9 | M | 0 | N | N |
| 10 | F | 0.20 | Y | N |
| 11 | M | 0.05 | Y | N |
| 12 | F | 0 | N | N |
| 13 | F | 0.05 | Y | N |
| 14 | M | 0 | Y | Y |
| 15 | M | 0.12 | Y | N |
| 16 | M | 0.15 | Y | Y |
| 17 | F | 0.04 | N | N |
| 18 | M | 0 | Y | N |
| 19 | M | 0 | Y | Y |
| 20 | F | 0 | N | N |
| 21 | F | 0 | N | N |
| 22 | M | 0 | N | N |
| 23 | M | 0.18 | N | N |
| 24 | M | 0 | N | N |
| 25 | M | 0 | N | Y |
| 26 | M | 0.07 | Y | N |
| 27 | F | 0 | Y | N |
| 28 | F | 0 | N | N |
| 29 | M | 0 | N | N |
| 30 | M | 0 | N | N |
| 31 | F | 0 | N | N |
| AF: Atrial fibrillation; µg/kg/min: microgram per kilogram per minute; M: Male; F: Female; Y:Yes; N:No | | | | |
