# Supplementary material for: Comparing volume-clamp method and intra-arterial blood pressure measurements in patients with atrial fibrillation admitted to the intensive or medium care unit
Source: J Clin Monit Comput. 2017 Jul 7;32(3):439–46. doi: 10.1007/s10877-017-0044-9 (PMC5943389; doi:10.1007/s10877-017-0044-9)
Supplement: Supplementary file 4 — Supplementary material 4 (DOCX 17 KB) [file 10877_2017_44_MOESM4_ESM.docx]

| **Supplemental table 4: Average after data analyses per patient. VCM vs computed arterial brachialis blood pressure measurements.** | | | | | | | | | | |
| --- | --- | --- | --- | --- | --- | --- | --- | --- | --- | --- |
|  |  |  |  |  |  |  |  |  |  |  |
|  | **Atrial fibrillation** | | | | | **Sinus rhythm** | | | | |
|  | **VCM** | **Intra-arterial** | **Mean difference** | **r²** | **ICC** | **VCM** | **Intra-arterial** | **Mean difference** | **r²** | **ICC** |
| Systolic blood pressure (mmHg) | 111 (21) | 114 (18) | -3 (11) | 0.71 | 0.81 | 114 (22) | 119 (21) | -5 (12) | 0.54 | 0.68 |
| Diastolic blood pressure (mmHg) | 65 (9) | 63 (9) | 3 (6) | 0.72 | 0.81 | 68 (9) | 64 (11) | 4 (5) | 0.51 | 0.64 |
| Mean blood pressure (mmHg) | 81 (12) | 81 (12) | 1 (7) | 0.71 | 0.81 | 84 (13) | 83 (14) | 1 (5) | 0.55 | 0.69 |
| Absolute beat-to-beat systolic blood pressure difference (mmHg) | 6 (3) | 7 (4) | 2 (2) | 0.81 | 0.82 | 3 (2) | 4 (3) | 2 (2) | 0.64 | 0.64 |
| *All data are displayed as mean (SD)*. VCM: Volume-clamp method; r: Correlation coefficient; ICC: Intra-class correlation | | | | | | | | | | |
